# Preclinical assessment of a novel small molecule inhibitor of tryptophan 2,3-dioxygenase 2 (TDO2)

**DOI:** 10.1186/2051-1426-2-S3-P196

**Published:** 2014-11-06

**Authors:** Stefano Crosignani, Gregory Driessens, Michel Detheux, Benoît Van den Eynde, Sandra Cauwenberghs

**Affiliations:** 1 iTeos Therapeutics, Gosselies, Belgium; 2 Ludwig Institute for Cancer Research (LICR, Brussels Branch) and de Duve Institute, Université catholique de Louvain (UCL), Brussels, Belgium

## Introduction

iTeos leverages the science of LICR to target metabolism of the tumor microenvironment and develops small molecule immunomodulators. TDO2 is a heme-containing enzyme highly expressed in the liver, in specific brain regions and liver/brain tumors. It catalyses the first and rate-limiting step of tryptophan catabolism along the kynurenine pathway and thereby regulates systemic tryptophan levels. Tryptophan (Trp) degradation by TDO2 suppresses anti-tumor immune responses and promotes tumor cell survival.

## Methods/results

Medicinal chemistry optimization following a primary HTS (98,000 compound library) led to the selection of three distinct chemical series. Compounds from all series inhibit kynurenine production with comparable nM potency compared to the earlier described Welcome compound 680C91 in cellular assays (IC_50 _190-450nM (A172)) and without activity on IDO1 (>100-fold selectivity for TDO2). Compounds with low clearance and half-lives of >1.5h in mice were identified for each of the three series. A compound was selected for further *in vivo *work due to its high oral bioavailability and long half-life in mice (3.5h). TDO2 inhibition was followed by tryptophan quantification in the blood (vehicle 21.0 ± 1.1 versus TDO2_i _(60mg/kg) 33.9 ± 1.51 μg/mL Trp; n = 10 mice/group). In a time-course experiment, inhibition of tryptophan degradation lasted up to 16h with a dose of 60mg/kg consistent with good rodent half-life. TDO2 inhibition in a P815 Mastocytoma tumor model overexpressing TDO2 showed efficacy with the investigational TDO2_i_, LM10. Further testing with an iTeos TDO2 compound with good PK/PD profile is ongoing on liver tumor models (HCC, liver metastases) as well as glioblastoma. A proprietary antibody for hTDO2 was generated to screen 15 different human cancer types (60 samples each) for TDO2 protein expression. Differential quantification will allow selection of putative clinical indications for TDO2 treatment in stand-alone or combination with standard of care treatment, vaccination or immune checkpoint inhibition.

## Conclusions

iTeos' drug discovery efforts have generated several scaffold structures for first-in-class TDO2 small molecule inhibitors suitable for *in vitro *and *in vivo *validation.

